# Oral Chronic Toxicity of the Safe Tetrodotoxin Dose Proposed by the European Food Safety Authority and Its Additive Effect with Saxitoxin

**DOI:** 10.3390/toxins12050312

**Published:** 2020-05-09

**Authors:** Andrea Boente-Juncal, Paz Otero, Inés Rodríguez, Mercedes Camiña, Mercedes Rodriguez-Vieytes, Carmen Vale, Luis M. Botana

**Affiliations:** 1 Laboratorio de Farmacología, Farmacia e Tecnoloxía Farmacéutica, Universidade de Santiago de Compostela, Facultad de Veterinaria, Campus Universitario s/n, 27002 Lugo, Spain; andrea.boente.juncal@usc.es (A.B.-J.); paz.otero@usc.es (P.O.); 2 Laboratorios Cifga, Benigno Rivera, 56, 27003 Lugo, Spain; inesrguez@cifga.es; 3 Departamento de Fisiología, Facultad de Veterinaria, Universidad de Santiago de Compostela, 27002 Lugo, Spain; merchi.camina@usc.es (M.C.); mmercedes.rodriguez@usc.es (M.R.-V.)

**Keywords:** Tetrodotoxin, saxitoxin, risk assessment, oral chronic toxicity, mollusk

## Abstract

Tetrodotoxin (TTX) is a potent natural toxin causative of human food intoxications that shares its mechanism of action with the paralytic shellfish toxin saxitoxin (STX). Both toxins act as potent blockers of voltage-gated sodium channels. Although human intoxications by TTX were initially described in Japan, nowadays increasing concern about the regulation of this toxin in Europe has emerged due to its detection in fish and mollusks captured in European waters. Currently, TTX is only regularly monitored in Dutch fishery products. However, the European Food Safety Authority (EFSA) has established a safety level of 44 µg/kg TTX as the amount of toxin that did not cause adverse effects in humans. This level was extrapolated considering initial data on its acute oral toxicity and EFSA remarked the need for chronic toxicity studies to further reduce the uncertainty of future toxin regulations. Thus, in this work, we evaluated the oral chronic toxicity of TTX using the safety levels initially recommended by EFSA in order to exclude potential human health risks associated with the worldwide expanding presence of TTX. Using internationally recommended guidelines for the assessment of oral chronic toxicity, the data provided here support the proposed safety level for TTX as low enough to prevent human adverse effects of TTX even after chronic daily exposure to the toxin. However, the combination of TTX with STX at doses above the maximal exposure level of 5.3 µg/kg body weight derived by EFSA increased the lethality of TTX, thus confirming that both TTX and paralytic shellfish toxins should be taken into account to assess human health risks.

## 1. Introduction

Tetrodotoxin is a potent sodium channel blocker responsible for human intoxications after ingestion of contaminated food. TTX was initially associated mainly with human fatalities occurring in South East Asia [1] but it has been demonstrated to affect also other regions in the Pacific and Mediterranean [2,3]. In fact, a severe case of human intoxication by consumption of pufferfish liver during the preceding 4 h prior to presentation, together with ingestion of canned foods and cocaine during the previous 3 days has been recently reported in Florida [4]. In Europe, the first non-fatal human intoxications by TTX were reported after the ingestion of a *Charonia lampas* trumpet shellfish captured in the Portuguese coast and purchased in Spain [5]. In this case, both TTX and its analogue 5,6,11-trideoxyTTX were identified in the digestive tract of the shellfish and in the urine and plasma of the intoxicated patient. As a result of the ingestion of the trumpet shellfish, the patient suffered general paralysis and required mechanical ventilation during 52 h after the intoxication, recovering 72 h after hospital admission [6,7]. In fact, recently, the presence of TTX in juvenile pufferfish *Lagocephalus sceleratus,* previously considered non-toxic, has been reported in European coasts, mainly in the Mediterranean Sea [2,8] with some of the tissues containing TTXs amounts that reached 2 mg/kg [9]. Furthermore, TTX has been also reported in mussels, oysters, and clams harvested in UK, Greece, and the Netherlands [3,10,11]. The ubiquitous detection of TTX in mollusks from the north part of Spain has been linked to the existence of high amounts in *Vibrio*, in particular *Vibrio parahaemolyticus* [12] but accumulation of TTX appears not to be a risk in Portuguese mollusks [13]. Recently, the presence of high amounts of TTX together with paralytic shellfish toxins (PSTs) has been reported in native pufferfish captured in European waters [14] and in Italian shellfish [15]. PSTs are a regulated group of toxins common in Europe, and saxitoxin (STX) is the representative toxin of this group [16]. Both TTX and STX share a common cellular target and are potent blockers of voltage-gated sodium channels (Na_v_). In spite of the toxicological lacks describing the potential toxic synergy between both groups of toxins, only one report has described the same acute toxicity for both TTX and STX in mice by feeding [17].

The current European legislation prevents the marketing of fishery products derived from poisonous fish known to accumulate TTX, specifically fish of the *Tetraodontidae*, *Molidae*, *Diodontidae*, and *Canthigasteridae* families [18]. Although TTX and its analogues are not currently regulated nor monitored in the European Union besides in the Netherlands [19], a safety level of 44 µg/kg TTX has been established by EFSA as the amount of toxin that did not cause adverse effects in humans [10,20]. The safety level for TTX has encouraged the Netherlands to include TTX in the shellfish monitoring program for the first time in Europe. [19]. In the Netherlands, a three year monitoring program (from 2015–2017) yielded TTX values as high as 253 µg TTX/kg in oysters and 101 µg TTX/kg in mussels in the year 2016 [19]. In view of the presence of TTX in the Netherlands, Dutch authorities established an initial safety limit of 20 µg TTX/kg in shellfish that was later modified after the EFSA assessment and finally established at 44 µg TTX/kg shellfish. Moreover, an acute reference dose (ARfD) for TTX of 0.25 µg/kg body weight (b.w.) was derived by EFSA following the assessment of the effects of the toxin in mice [19,20]. The ARfD established by EFSA was determined on the basis of the oral acute lethal toxicity in mice that yielded a lethal dose 50 (LD_50_) of 232 µg TTX/kg body weight while the dose of 25 µg/kg b.w. had non-observed adverse effects [21]. However, animals treated orally with a single TTX dose from 125 to 500 µg/kg b.w. showed liquid and gas accumulation in the stomach while ultrastructural changes were observed in the spleen, liver and intestine two hours after oral administration of TTX to mice at doses of 500 µg/kg [22]. Recently, the first report on the chronic toxicity of TTX after exposure of mice to low oral doses of toxin following the internationally adopted guidelines of the Organisation for Economic Co-operation and Development (OECD) has been released [23]. In this report, 28 days of repeated oral exposure of female Swiss mice to TTX doses ranging from 25 to 125 µg/kg, indicated that the toxin suppressed urine production in mice dosed with 75 and 125 µg/kg and altered blood biochemical parameters as well as the urinalysis. Additionally, ultrastructural alterations in the kidney and heart of mice after a 28-daily oral ingestion of TTX at 125 µg/kg were also observed [23]. As a result of the significant impact of this preliminary data, it is necessary to have an initial approach to the chronic toxicity of TTX and its analogues considering the reference TTX dose recently reported by EFSA. Furthermore, for STXs a regulatory level of 800 µg/kg of STX and analogues was established by EFSA and this level represents an intake of 320 µg of toxin, which corresponds to 5.3 µg/kg b.w. in a 60 kg adult [24]. As recognized by EFSA, human consumption of this amount of toxin could be a threat to health since it is higher than the acute reference dose (ARfD) of 0.5 µg STX equivalents /kg b.w. which correspond to 30 µg STX equivalents in each portion for a 60 kg adult [24]. Therefore, the main aim of this work was to analyze the chronic effects of TTX following the international guidelines of the OECD [25] and using the dose reported as safe by EFSA, alone or in combination with increasing STX amounts. 

Both, TTX and the marine toxins saxitoxins are potent neurotoxins that share a common mechanism of action acting as reversible pore blockers at site I of voltage-gated sodium channels [26] and, as far as we know, this is the first report evaluating the oral chronic effects of the safety level of the TTX dose established by EFSA and the chronic oral toxicity of the combination of STXs and TTX following internationally adopted guidelines.

## 2. Results

### 2.1. Quantification of the Commercial TTX Employed for In Vivo Experiments

**Figure 1:**
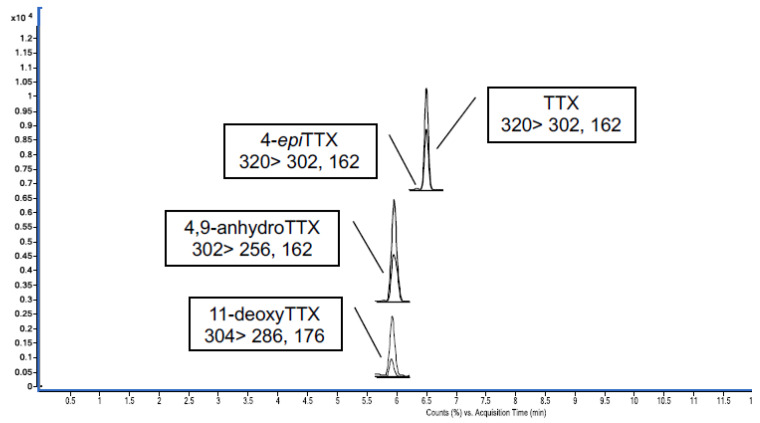
UHPLC-MS/MS chromatogram obtained by multiple reaction monitoring (MRM) mode of certified reference material-tetrodotoxin (CRM-TTX) from CIFGA.

**Figure 2:**
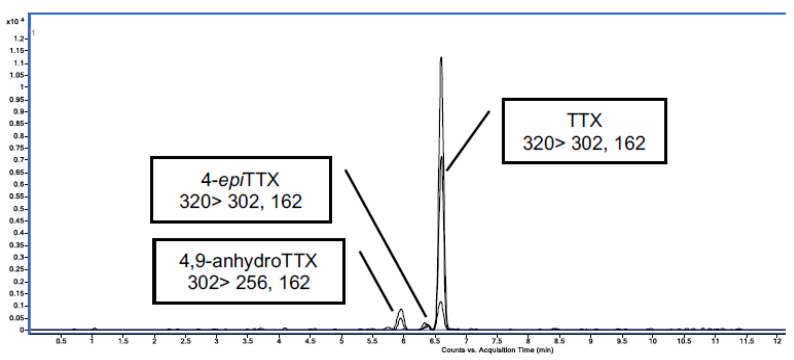
UHPLC-MS/MS chromatogram obtained by MRM mode of the TTX from Tocris.

In this work, commercial TTX from Tocris was employed for the in vivo experiments. Firstly, the amount of TTX in the standard was quantified against a certified reference material (CRM TTX) purchased from CIFGA. TTX from Tocris was dissolved in acetic acid (AcOH) 1 mM following the manufacturer instruction and the Tocris stock solution was quantified against the CRM TTX that contained 25.9 ± 1.3 µg TTX/g (Figure 1). The TTX analogues present in the Tocris standard were detected and quantified by LC-MS/MS (Figure 2 and Table 1). The calibration curve to establish the chemical purity of TTX from Tocris was performed with the CRM TTX standard from CIFGA assuming that related analogues would give a response similar to TTX. Quantification of the toxin and its analogues was realized using multiple reaction monitoring (MRM) acquisition in positive mode (Table 1). A six-point calibration curve ranging from 7.39 ng/g to 121.53 ng/g was used (R^2^ = 0.999). The limit of detection (LOD) was 0.78 ng/g and the limit of quantification (LOQ) 2.60 ng/g [27]. 

Tocris TTX standard quantification showed that the sample contained 96% of TTX, 3% of 4,9-anhydroTTX and 1% of 4-*epi*TTX. Taking into account the toxicity equivalence factor (TEF) of this group of toxins the amount of TTX analogues, 4-*epi*TTX and 4,9-anhydroTTX, was negligible compared to TTX [28]. Therefore, these analogues were not considered when the in vivo dose was calculated.

### 2.2. TTX at the Dose of 44 µg/kg did not Elicit Changes in Body Weight, Food Intake or Urine Production after the 28 Days Oral Administration Period

To evaluate the potential chronic oral toxicity of TTX, female Swiss mice were fed daily by gavage, for a period of 28 days with the toxin at doses of 44 and 20 µg/kg. These doses were selected on the basis of the acute reference dose proposed by EFSA and the regulatory limits adopted initially by the Dutch food Authorities [19,20]. The same procedure was performed in control mice, which received only the same volume of toxin solvent.

Animals fed daily with 44 µg/kg of TTX survived the entire experimental period and did not exhibit any behavioral alteration or toxicity signs nor change in body weight. Table 2 summarizes the mean body weights and food intake (both parameters registered weekly) during the 28 days treatment period.

### 2.3. TTX at the Dose of 44 µg/kg Altered Blood Biochemical Parameters

**Figure 3:**
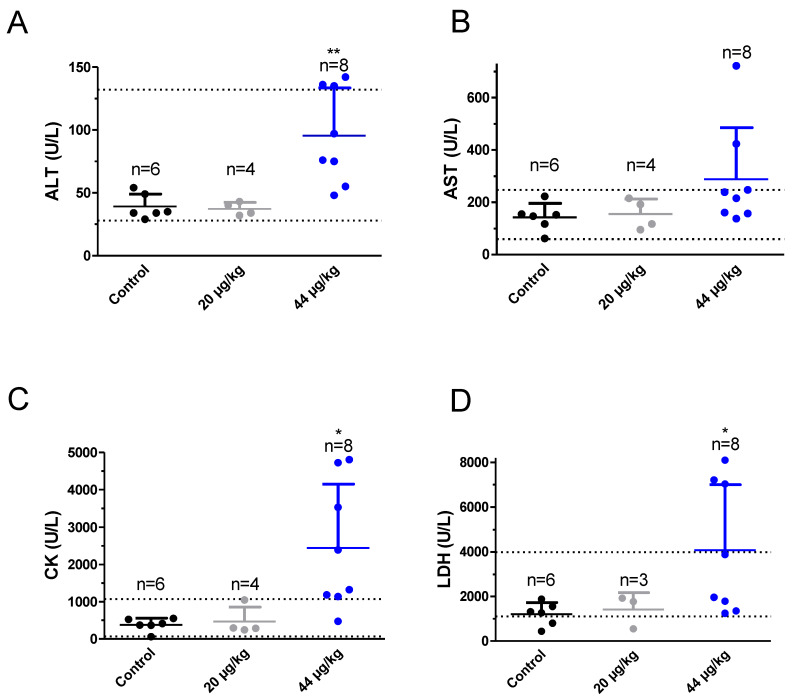
Blood levels of alanine transaminase (ALT) (**A**), aspartate transaminase (AST) (**B**), creatine kinase (CK) (**C**), and lactate dehydrogenase (LDH) (**D**) in control Swiss female mice and in mice dosed daily by gavage with TTX at 20 µg/kg or 44 µg/kg. * *p* < 0.05; ** *p* < 0.01 versus control mice.

At the end of the 28 days of treatment, mice were euthanized and biochemical analysis of blood, extracted by cardiac puncture immediately after euthanasia, was performed in samples from control and TTX-treated mice (Figure 3). Blood parameters analyzed included alanine transaminase (ALT), aspartate transaminase (AST), lactate dehydrogenase (LDH), blood urea nitrogen (BUN), creatine kinase (CK) and electrolyte levels (Cl^−^, Na^+^ and K^+^). As shown in Figure 3A, mean ALT values were 39 ± 4 U/L (*n* = 6) in control animals, 37 ± 3 U/L in four animals dosed with 20 µg/kg of TTX and 96 ± 13 U/L (*n* = 8) in mice dosed with 44 µg/kg of TTX. One-way analysis of variance followed by Dunnett’s post hoc test showed significant differences between ALT levels in control animals and in mice fed orally with TTX at 44 µg/kg (t = 3.5178; df (Degrees of freedom) = 12; *p* < 0.0042) but the levels of ALT in both groups were within the normal reference range (28–132 U/L for the IDEXX). In contrast, for AST (Figure 3B) mean values were 143 ± 22 U/L (*n* = 6) in control mice, 154 ± 28 (*n* = 4) in mice dosed daily with 20 µg/kg TTX and 288 ± 69 U/L (*n* = 8) in mice fed with 44 µg/kg of TTX. Statistical analysis of these data did not yield significant differences between the control group and the TTX-treated animals. Creatine kinase blood levels (Figure 3C), were 383 ± 72 U/L for the control group (*n* = 6), 467 ± 190 U/L (*n* = 4) for the group of animals dosed with TTX at 20 µg/kg and 2400 ± 600 U/L (*n* = 8) for the mice fed with TTX at 44 µg/kg. Statistical comparison of these data showed significant differences between control animals and animals treated with TTX at 44 µg/kg (t = 2.919; df = 12; *p* = 0.0129). However, in this case, the blood level of CK in the animals dosed daily with 44 µg/kg TTX was well above the IDEXX reference range which is between 68 and 1070 U/L, with 7 out of 8 TTX-treated animals showing CK levels above 1070 U/L. The following blood parameter evaluated was lactate dehydrogenase (Figure 3D) and in this case blood levels were 1200 ± 210 U/L (*n* = 6) in control animals, 1420 ± 440 (*n* = 3) in mice fed with 20 µg/kg TTX and 4070 ± 1040 U/L (*n* = 8) in the group of animals that received 44 µg/kg TTX daily (Figure 3D). Again, in this case, statistically significant differences were observed between control animals and those that received 44 µg/kg TTX (t = 2.344; df = 12; *p* = 0.0371) and LDH values in TTX treated animals were slightly higher than the IDEXX reference range for blood LDH levels which is between 1105 and 3993 U/L, however in this case only three out of the 8 mice showed LDH levels that duplicated the higher reference level while the other mice gave normal blood LDH levels. In the case of BUN, for control animals mean blood levels where 22 ± 1 mg/dl (*n* = 6), 16 ± 1 mg/dl (*n* = 4) in animals dosed daily with 20 µg/kg TTX and 15 ± 1 mg/dl (*n* = 7) in animals that received 44 µg/kg of TTX per day. A significant decrease of BUN in mice feed daily with 20 µg/kg or 44 µg/kg TTX was found. However, no changes in BUN levels were observed previously in a similar study using TTX at doses of 75 µg/kg [23].

**Figure 4:**
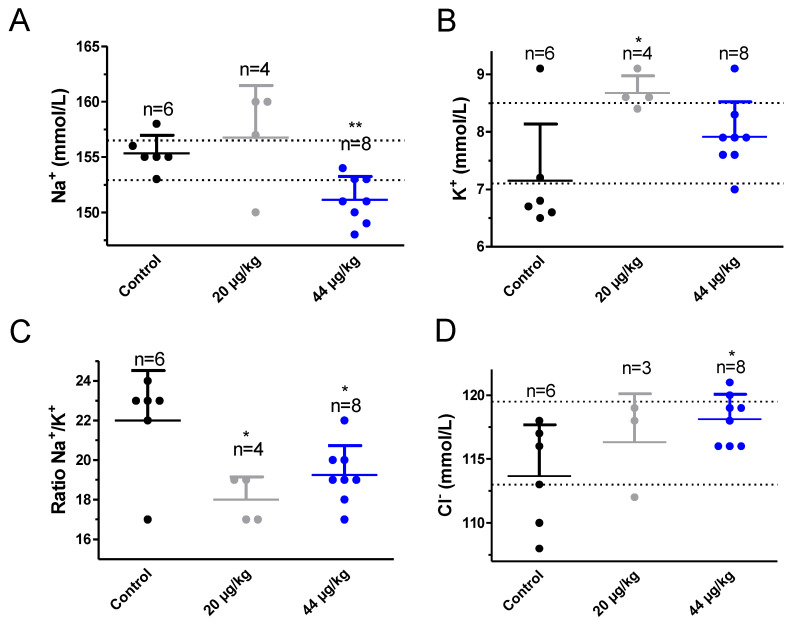
Blood levels of sodium (**A**), potassium (**B**), ratio sodium/potassium (**C**) and chloride (**D**) in control Swiss female mice and in mice dosed daily by gavage, over a 28 day period, with TTX at 20 µg/kg or 44 µg/kg. * *p* < 0.05; ** *p* < 0.01 versus control mice.

Next, the effect of daily TTX treatment on blood electrolyte levels was evaluated (Figure 4). As shown in Figure 4A at the dose of 20 µg/kg, TTX did not affect blood sodium levels but at the dose of 44 µg/kg the toxin slightly decreased blood sodium from 155 ± 1 mmol/L in control animals (*n* = 6) to 151 ± 1 mmol/L (*n* = 8) in toxin-treated animals and this difference was statistically significant (t = 4.060; df = 12; *p* = 0.0016). When blood potassium levels were evaluated statistically significant differences were found between control animals and animals treated with 20 µg/kg TTX. Thus, blood potassium levels were 7.2 ± 0.4 mmol/L (*n* = 6) in control mice, 8.6 ± 0.1 mmol/L (*n* = 4) in mice fed daily with 20 µg/kg TTX (t = 2.952; df = 8; *p* = 0.0184) and 7.9 ± 0.2 mmol/L in mice dosed with 44 µg/kg of TTX (t = 1.793; df = 12; *p* = 0.0983). As a consequence, the Na^+^/K^+^ ratio was significantly decreased from 22 ± 1 in control animals to 18 ± 1 in mice fed daily with 20 µg/kg TTX (t = 2.921; df = 8; *p* = 0.0193) and 20 ± 1 in mice fed daily with 44 µg/kg TTX (t = 2.242; df = 11; *p* = 0.0466). When blood chloride levels were analyzed, a significant rise in blood chloride was found in mice treated daily with TTX at 44 µg/kg (Figure 4D). Blood chloride levels were 113 ± 2 mmol/L (*n* = 6) in control animals, 116 ± 2 mmol/L (*n* = 3) after treatment with 20 µg/kg TTX and 118 ± 1 mmol/L (*n* = 8) in mice treated daily with TTX at 44 µg/kg, and this effect was statistically significant (t = 2.749; df = 12; *p* = 0.0176).

### 2.4. Daily Feeding of Mice with TTX at Doses of 20 µg/kg did not Affect Urine Parameters but at 44 µg/kg Blood in Urine and Increased Bilirubin Were Present

We have recently reported that exposure of mice to TTX at doses of 125 µg/kg diminished urine production and altered some urine parameters [23]. Therefore, in order to complete the safety evaluation of the chronic effects of TTX at doses of 20 µg/kg and 44 µg/kg, urine parameters were evaluated during the 24 h before sacrifice, after mouse placement in individual metabolic cages. Urine parameters evaluated were color, clarity, specific gravity, protein, glucose, ketones, blood/hemoglobin, bilirubin, and urobilinogen. Urine parameters in control and TTX-treated mice are summarized in Table 3. No statistically significant differences were found between control mice and animals dosed daily with 20 µg/kg TTX or with 44 µg/kg TTX. Blood and bilirubin were present in urine. In this case, most of the urine parameters evaluated were normal for all the mice, however, in four animals dosed with TTX at 44 µg/kg, high bilirubin levels, as well as presence of blood in urine, were detected.

### 2.5. Quantification of TTX in Blood, Urine, Feces, and Organs by UHPLC-MS/MS

**Figure 5:**
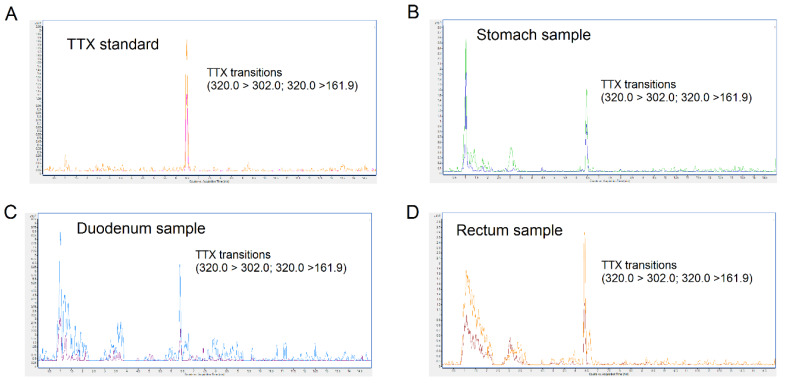
UHPLC-MS/MS chromatograms of TTX standard (**A**), stomach sample (**B**), duodenum sample (**C**), and rectum sample (**D**) collected 1 h after feeding mice with TTX at a dose of 44 µg/kg. “Y” axis represent counts and the “x” axis represent the acquisition time in minutes

**Figure 6:**
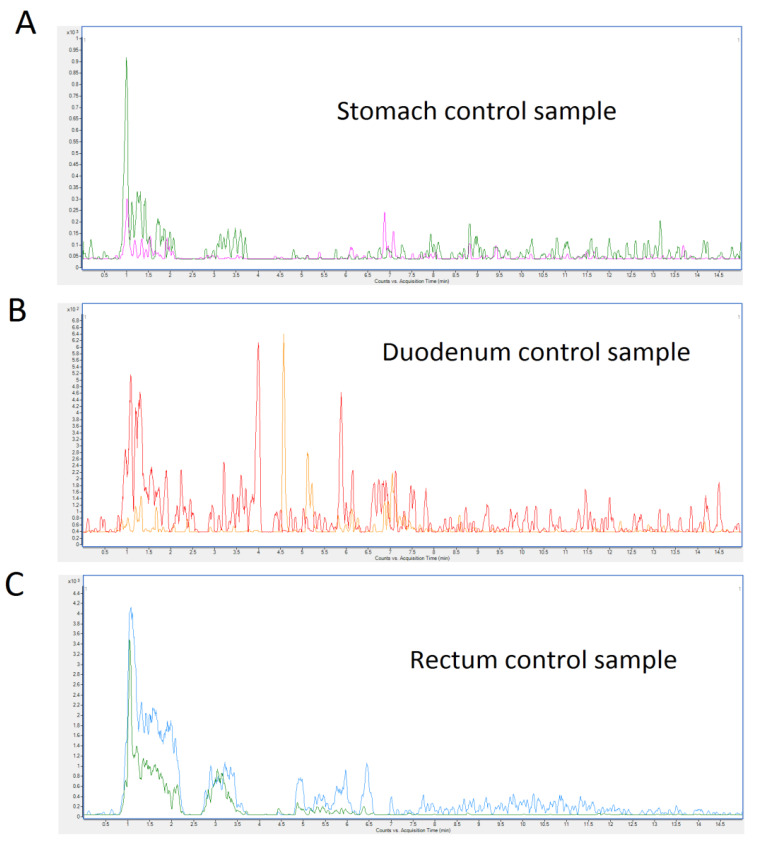
UHPLC-MS/MS chromatograms of control mice. (**A**), stomach sample (**B**), duodenum sample (**C**) rectum sample. Control mice were fed for 28 days with toxin solvent.

On the day of sacrifice mice, with free access to food and water, were fed with the toxin at 44 µg/kg and, one hour after toxin administration, animals were euthanized. After sacrifice, different organ samples were collected for toxin quantification. The quantity of TTX detected in biological samples is summarized in Table 4. TTX was detected in stomach, duodenum, and rectum samples. A representative chromatogram of each sample from TTX-treated mice is shown in Figure 5. Samples of heart, lung, liver, kidney, spleen, urine and blood did not show TTX. No TTX analogues were detected in the biological samples. Representative chromatograms of control mice are shown in Figure 6.

### 2.6. Combined Chronic oral Toxicity of TTX and Saxitoxin Mixtures

Once evaluated the chronic toxicity of TTX, the simultaneous toxicity of the safety reference dose of TTX (44 µg/kg) was evaluated in combination with low STX doses. The lower STX dose chosen was 5.3 µg/kg as the maximum intake extrapolated by EFSA [24] and this dose was increased by a factor of 3.2 as recommended by the OECD guidelines [25]. Thus, mice received daily by gavage either 44 µg/kg TTX in combination with 5.3 µg/kg STX or the same TTX dose plus 17 µg/kg or 54 µg/kg STX. Four mice were used as control groups and for the dose of TTX + 5.3 µg/kg STX and five mice for the two higher STX doses. No mortality was observed at the dose of TTX + 17 µg/kg STX doses but at the dose of TTX + 5.3 µg/kg STX one animal died on day 22 of treatment after unforeseen convulsions. At the dose of 44 µg/kg TTX + 54 µg/kg STX three out of five mice died on day 2 and day 11 of treatment after sudden convulsions and rapid death while the other three mice survived the 28 day administration period. No effect of the mixtures on body weight was observed as indicated in Table 5.

Similarly, no changes in food consumption and feces or urine production were observed between control and treated mice when evaluated for 24 h in metabolic cages at the end of the treatment (Table 6).

### 2.7. TTX at the Dose of 44 µg/kg Combined with STX did not Alter Blood Biochemical Parameters

**Figure 7:**
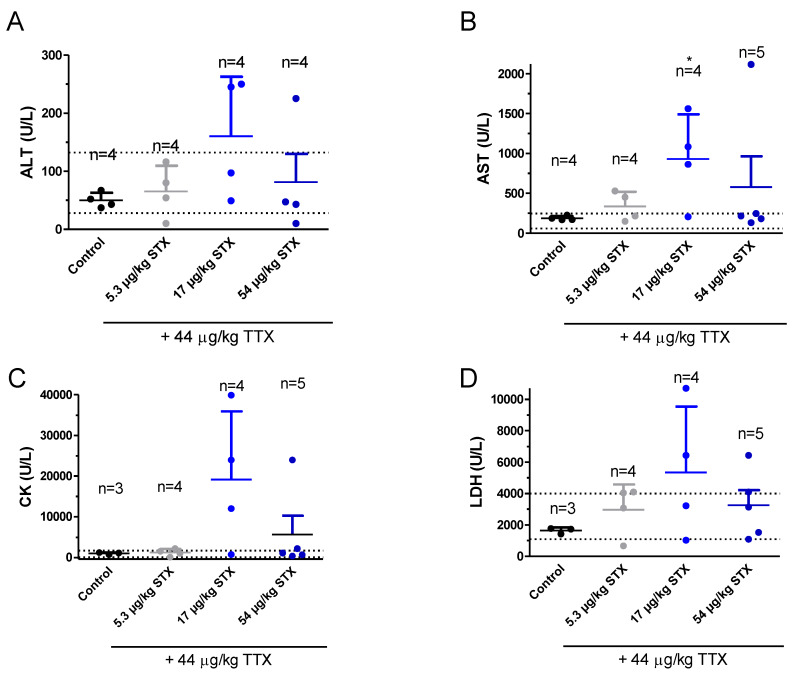
Blood levels of ALT (**A**), AST (**B**), CK (**C**), and LDH (**D**) in control Swiss female mice and in mice dosed daily by gavage with the combination of TTX at 44 µg/kg and STX at 5.3, 17, or 54 µg/kg. * *p* < 0.05 versus control mice.

At the end of the 28 days of treatment, mice were euthanized and, as mentioned above, biochemical analysis of blood, extracted by cardiac puncture immediately after euthanasia, was performed in samples from control and TTX-treated mice (Figure 7). However, in these groups of mice the amount of blood did not allow to evaluate blood electrolyte levels in a sufficient number of animals, thus only ALT, AST, CK, and LDH levels are reported. As shown in Figure 7A, blood ALT levels (U/L) were 50 ± 7 for control animals (*n* = 4), 65 ± 20 for TTX combined with 5.3 µg/kg STX, (*n* = 4), 160 ± 50 (*n* = 4) for TTX combined with 17 µg/kg STX and 87 ± 50 (*n* = 4) TTX + 54 μg/kg STX. Therefore, chronic STX and TTX administration to mice did not affect blood ALT levels. Blood AST levels (U/L) were 190 ± 14 (*n* = 4) in controls, 337 ± 9 (*n* = 4) in animals dosed with TTX and 5.3 µg/kg STX, 930 ± 280 (*n* = 4) at the dose of 17 µg/kg STX and 44 µg/kg TTX (*p* < 0.05; t = 2.628; df = 6) and 580 ± 380 (*n* = 5) in animals fed with TTX + 54 µg/kg STX. These slightly altered values of TTX correspond to two mice that had ALT levels above the reference range in the group of TTX + 17 µg/kg STX and to one mouse with increased AST when TTX was administered with 54 µg/kg STX (Figure 7B). CK levels were neither statistically significantly different between control mice and animals treated with the combination of TTX and different STX doses (Figure 7C). However, in the group of TTX and 17 µg/kg STX levels three out of four animals had CK levels about ten times higher than control animals while in the TTX + 54 µg/kg STX only one animal had high CK levels. As shown in Figure 7D, LDH levels did not differ significantly between control animals and animals treated with the combination of TTX and STX. In this case LDH levels (U/L) were 1500 ± 140 (*n* = 4) in control animals, 2970 ± 800 (*n* = 4) in animals doses daily with TTX and 5.3 µg/kg STX, 5300 ± 2100 (*n* = 4) in animals treated with TTX + 17 µg/kg STX and 3200 ± 960 (*n* = 5) for the group of mice fed with TTX + 54 µg/kg STX.

## 3. Discussion

TTX is an emerging toxin in mollusks and fish caught in European waters [10,29,30,31]. The presence of TTX and regulated marine toxins such as the paralytic shellfish toxins saxitoxins in pufferfish from the Atlantic has been recently reported [14]. Furthermore, recent reports have described the presence of this potent neurotoxin in mollusks and gastropods worldwide [3,5,6,8,11,28,32] raising growing concerns about TTX regulation in fish and mollusks [10,20]. Recently EFSA reported a mean human exposure level for TTX after clams, mussels, or oysters ingestion of 0.02, 0.03, and 0.09 µg/kg b.w., respectively, whereas for the other bivalves the level of acute exposure to TTX was zero [20]. The highest 95th percentile exposure was estimated for oysters, being 0.08 µg/kg b.w. [20]. However, in the same report, the need to evaluate the chronic effects of the toxin was highlighted. Therefore, this work attempted to evaluate the chronic effects of TTX following the principles of the internationally adopted OECD guidelines for the 28 days exposure period, as adapted to comply with the principle of reduction required by the Directive 2010/63/EU [25] and using the safety TTX levels proposed by EFSA. Even when this level of exposure is unlikely to occur in humans, the data provided in this work further supports future legislations on the levels of TTX in marine fishery products and provide the first data on the chronic effect of TTX at the safety level already adopted for some European countries. The data presented here indicated that after a 28-day dosage of mice with TTX at doses of 44 and 20 µg/kg b.w. only increases in creatine kinase blood levels, decrease in blood sodium levels, increase in potassium and chloride levels and decrease in the blood sodium/potassium ratios, were observed. All mice were healthy after chronic administration of the toxin by gavage and did not show any signs of apathy or lethargy during the treatment period. Using a large portion size of 400 mg/kg EFSA concluded that a concentration below 0.044 mg TTX equivalents/kg of shellfish meat did not have adverse effects in humans. However, the same scientific opinion remarked the lack of data on the chronic effects of this toxin [20]. In this work, this recommendation has been addressed evaluating the chronic effects of TTX in mice after daily oral administration of the toxin by gavage, following internationally adopted guidelines [25].

In contrast to chronic neosaxitoxin administered either intraperitoneally or intramuscularly [33], at the doses evaluated, TTX affected neither food intake nor urine or feces production or body weight. In this regard, pharmacokinetic differences among the distinct routes of administration may account for these differences since the oral route will decrease TTX absorption and increase TTX metabolism. According to this, a much higher LD_50_ has been described for TTX after oral administration in contrast with the intraperitoneal route [20]. The only effects observed after the daily administration of TTX at doses of 44 µg/kg were an increase in the blood creatine kinase and lactate dehydrogenase levels, a small decrease in blood sodium concentration, and an increase in blood chloride and potassium levels. Although previous work has reported cardiovascular alterations after human intoxications by TTX [32], several initial reports extensively reviewed [34] have described broad cardiovascular effects of TTX in mice and other animal species at doses ranging from 8 to 10 μg/kg applied either by the intravenous or the intraperitoneal route. Albeit these initial studies excluded a direct action of TTX on the heart, hypotension and bradycardia were usually observed while a reduced contractile force was observed in some studies after TTX injection to different animal species (reviewed in [34]). Cardiovascular alterations are also well documented in human intoxications by TTX [35,36,37] and cardiovascular/pulmonary alterations including hypotension or hypertension; vasomotor blockade; cardiac arrhythmias with sinus bradycardia, asystole, tachycardia, and atrioventricular node conduction abnormalities have been described in third grade patients intoxicated by this toxin [38]. Nevertheless, creatine kinase elevations in blood of human patients intoxicated with paralytic shellfish toxins have been previously described [39], even though the increase in this enzyme did not represent the severity of the human illness. Multiple factors, including hypertension may give rise to a blood increase in creatine kinase levels [40], however, although blood pressure was not measured in this work, this effect is unlikely to occur in animals due to the prevalent hypotension observed in several works after TTX administration.

Noteworthy, combinations of different toxins should be considered for public health purposes. Consumption of shellfish contaminated with different toxins could be possible and especially important if they share a common cellular target such as TTX and STX, both potent blockers of voltage-gated sodium channels (Nav) [16]. Likewise, TTX and STX could be present in native species of pufferfish from the NE Atlantic reaching high concentrations [14] and in Italian shellfish [15]. The synergy between both groups of toxins was reported showing that the oral toxicity of TTX by feeding was the same as that of STX and that the toxicities of the two toxin types are additive [17]. Nevertheless, this is an acute toxicity study, and little is known of the potential impact these toxins may have on public health in the long term. 

As far as we know, this is the first report evaluating the oral chronic effects of the safety level of the TTX dose established by EFSA and the chronic oral toxicity of the combination of STXs and TTX following internationally adopted guidelines. Our present work examines the cumulative effects of TTX and STX oral chronic toxicity. The results presented here indicated that combination of 44 µg/kg TTX + 5.3 µg/kg STX leads dead after unforeseen convulsions on day 22 of treatment. Increasing STX dose to 54 µg/kg three out of five mice died on day 2 and day 11 of treatment after sudden convulsions and rapid death while the other three mice survived the 28 day administration period. These findings are consistent with the clinical signs of toxicity induced by STX and TTX where death is associated with jerky and running movements of the back legs [17]. However, animals fed daily with 44 µg/kg of TTX survived the entire experimental period and did not exhibit any behavioral alteration or toxicity signs nor change in body weight. The combination of TTX with STX at doses above the maximal exposure level of 5.3 µg/kg body weight derived by EFSA increased the lethality of TTX, thus confirming that both TTX and paralytic shellfish toxins should have potential additive effect.

Subtle alterations in blood biochemistry parameters such as an increase in LDH and CK levels were also identified in mice feed with TTX [23]. Again, the only effects observed after the daily administration of TTX at doses of 44 µg/kg were an increase in the blood creatine kinase and lactate dehydrogenase levels over physiological range. However, in the group of TTX + 17 µg/kg STX levels of ALT and AST were above normal physiological range, indicating again that the toxicities of TTX and STX are additive.

Altogether, the data presented here indicated that chronic oral exposure of mice to TTX, at the safety levels established by EFSA, did not appear to have major effects on animal behavior or biochemical blood parameters and furthermore, since this level of exposure is unlikely to occur in humans, the safety level of TTX in fishery products seems to be low enough to protect consumer´s health. Nevertheless, simultaneous presence of TTX and STX represents an additional concern for food fishery security since both toxins have been reported in several European countries in recent years. The results obtained here indicate potential harmful effects of safe concentration of TTX combined with STX that require further and detailed studies in order to reduce the human health risk of TTX and STX.

## 4. Materials and Methods

### 4.1. Toxins and Chemicals

Tetrodotoxin citrate was purchased either from Tocris (CAS Number 18660-81-6, Bristol, UK) with a molecular weight of 319.27 g/mol or as a certified reference material (CRM-TTX) from Laboratorio CIFGA S.A. (Lugo, Spain). Ampoules of CRM-TTX contained 0.5 mL of solution with a certified concentration of 25.9 ± 1.3 µg TTX/g (CAS Number 4368-28-9) and 2.99 ± 0.16 µg 4,9-anhydroTTX/g (CAS Number 13072-89-4) dissolved in aqueous AcOH. STX was supplied by CIFGA as a solution of saxitoxin dihydrochloride (CAS Number 35554-08-06 with a molecular weight of 372.21 g/mol), this certified reference material has a purity higher than 99%. STX at a concentration of 20.5 ± 1.0 µg/g was provided dissolved in HCl 3 mM (pH = 2.63). Acetonitrile was supplied by Panreac (Barcelona, Spain). All the solvents used were HPLC or analytical grade and the water was distilled and passed through a water purification system (Milli-Q, Millipore, Merck KGaA, Darmstadt, Germany). Formic acid was purchased from Merck (Darmstadt, Germany). Ammonium formate was from Fluka (Sigma-Aldrich, Merck KGaA, Darmstadt, Germany).

### 4.2. UHPLC-MS/MS Analysis

Chromatographic separation of TTX and its analogs was performed using a 1290 Infinity ultra-high-performance liquid chromatography system coupled to a 6460 Triple Quadrupole mass spectrometer (Agilent Technologies, Waldbronn, Germany). An ACQUITY UPLC BEH Amide column (2.1 × 100 mm, 1.7 µm, Waters,(Cerdanyola del Vallès, Barcelona, Spain) was employed to separate the toxins at 35 °C. The injection volume was 5 µl. Samples were held in the autosampler at 4 °C. Mobile phase A was 100% water with 0.1% formic acid and 10 mM ammonium formate. Mobile phase B was 98% MeCN supplemented with 0.1% formic acid and 2% 100 mM ammonium formate. The gradient program with a flow rate of 0.3 mL/min was started with 95% B and then a linear gradient to 5% B in 11 min. After a linear isocratic hold time of 1 min at 5% B the starting conditions of 95% B were re-established in 1 min. Finally, initial condition, 95% B, was kept 2 min until next injection [27]. Agilent source conditions were set to achieve the best sensitivity for all compounds and established as follow: drying gas temperature of 250 °C and flow of 11 L/min, nebulizer gas pressure of 55 psi, sheath gas temperature of 400 °C and flow of 12 L/min. The capillary voltage was set to 3000 V in positive mode with a nozzle voltage of 0 V. A nitrogen generator NITROMAT N-75 ECO from Worthington Creyssensac (Spain) was employed. The analysis was performed using electrospray ionization (ESI) and MRM acquisition in positive mode. The MassHunter Optimizer software was used to optimize fragmentor (Frag), cell accelerator voltage (CAV) and collision energy (CE). As shown in Table 7 two product ions were analyzed per compound, one for quantification and another for confirmation [27].

### 4.3. Sample Preparation for UHPLC-MS Analysis

TTX extraction from blood, feces, urine, and organs was realized following a previously described procedure [23]. Briefly, 100 µL of mice blood, extracted by cardiac puncture were mixed with 800 µL of 2% acetic acid and vortexed for 5 min, transferred to an ultrafiltration spin column and centrifuged at 4000 rpm for 40 min. Mouse feces (0.3 g) were mixed with 1600 µL of 2% acetic acid and vortexed for 5 min and the extract was centrifugated at 4000 rpm for 15 min. The supernatant was transferred to the ultrafiltration spin column and centrifuged again at 4000 rpm for 60 min. Mouse heart (0.150–0.225 g), lung (0.212–0.331 g), liver (0.750–0.925 g), kidney (0.284–0.493 g), spleen (0.192–0.249 g), stomach (0.429–0.546 g), duodenum (0.202–1.020 g), rectum (0.785–1.205 g) were mixed with 800 µL of 2% acetic acid and vortexed for 5 min. Afterwards, samples were extracted again with 800 µL of the same solution. Supernatants were pooled, filtered with 0.22 µm filters, transferred to an ultrafiltration spin column and centrifuged at 4000 rpm for 30 min. Urine (300 µL) was mixed with 1600 µL of 2% acetic acid and vortexed, placed in the ultrafiltration spin column and centrifuged at 4000 rpm for 60 min. Finally, all the ultrafiltrated solutions were dried, dissolved in 200 µL of 0.03 M acetic acid, and filtered again through a 0.22 µm filter before transfer to the LC-MS vial for analysis. The same protocol was applied to blood, feces, urine, and organs from control mice.

### 4.4. In Vivo Experimental Procedure

*In vivo* studies were performed with Swiss female mice initially weighing 19–25 g (4 weeks old at the beginning of the treatment). All animal procedures were performed according to the European legislation (EU directive 2010/63/EU) and Spanish legislation (Real Decreto 53/2013, Decreto 296/2008), and with the principles approved by the Institutional Animal Care Committee of the Universidad de Santiago de Compostela under procedure number 06/19/LU-002, authorized on 1 March 2019 (A12X00509). The toxin used in the in vivo experiments was from Tocris and contained 96% TTX, 3% 4,9-anhydroTTX and 1% 4-epiTTX. Immediately before feeding the animals, TTX was diluted in 0.9% saline solution to achieve each dose. Doses ranging from 20 to 44 µg/kg b.w. of TTX were administrated by gavage every 24 h over 28 days, dissolved in a final volume of 200 µL of solution per mouse. Control mice were fed with 200 µL of saline solution containing the corresponding amount of AcOH. Control and TTX-treated animals were observed intermittently during study and especially during the first two hours after dosage. Animals were housed with free access to food and water in separate cages, one cage per toxin dose and a maximum of 5 animals per cage. Finally, four additional groups of mice were fed consecutively with control toxin solvent, TTX at 44 µg/kg and STX at 5.3 µg/kg, or the combination of this dose of TTX with either 17 µg/kg STX or 54 µg/kg STX. Toxin doses were administrated sequentially, starting at the lower dose and initiating the following dosage period after finishing the first 28 day treatment. During treatment, mice were kept in rooms with constant temperature, humidity, and a controlled photoperiod at the animal facilities of the School of Veterinary Medicine of the University of Santiago de Compostela (Code: AE-LU-002). During experiments, animals were weighed weekly at day 0 (day of the fist treatment), day 7, day 14, day 21, and day 28, and food consumption was also registered at the same intervals. Euthanasia was performed on day 28. After the last TTX dosage (day 27), control and TTX-treated animals were placed in metabolic cages for 24 h in order to monitor urine and feces production. Finally, animals were euthanized in a CO_2_ chamber and immediately after death confirmation blood and tissue samples were collected. All animals in the study were subjected to a full necropsy, including detailed visualization of heart, liver, lungs, kidneys, spleen, stomach, duodenum, rectum, and cerebrum.

### 4.5. Blood and Urine Analysis

Blood was extracted by direct cardiac puncture in the ventricle of euthanized mice and transferred to lithium heparin microtubes (2.5 U.I. LH/mL), mixed for 30 s and centrifuged for 90 s in a high speed microcentrifuge (IDEXX Stat Spin 15,800 rpm/12,000× *g*, IDEXX Europe B.V., Hoofddorp, The Netherlands). Samples with altered visual properties such as high hemolysis, icterus, or lipidemia were not further processed. Blood analysis was performed using, the computer-controlled biochemical analyzer Catalyst Dx (IDEXX VetLab Station, IDEXX Europe B.V., Hoofddorp, the Netherlands). Briefly, 300 μL of plasma were used to evaluate electrolyte levels (Cl^−^, Na^+^ and K^+^), ALT, AST, LDH, CK, and BUN. All the biochemical parameters were first analyzed in undiluted samples but if the measured parameter was out of the analyzer reference range an automatic dilution with physiological saline was performed. For urine analysis, a reflectance photometer (IDEXX VetLab UA, Europe B.V., Hoofddorp, The Netherlands), which reads and evaluates IDEXX UA Stripsm was used to determine color, clarity, protein, glucose, ketones, blood hemoglobin, bilirubin, and urobilinogen. Urine specific gravity was measured with a refractometer. Due to the small amount of blood or urine obtained from some mice it was not possible to measure all the parameters for each animal.

## Figures and Tables

**Table 1:** TTXs analogues in the Tocris toxin quantified by MRM mode. The toxicity equivalence factors were obtained from [28].

TTX Analogues	Concentration (µg/g)	TEFs	µg TTX Equivalents/g
**TTX**	240.71	1	240.71
**4-*epi*TTX**	3.30	0.16	0.528
**4,9-anhydroTTX**	7.17	0.02	0.1434

**Table 2:** Daily exposure of mice to TTX at doses of 20 µg/kg and 44 µg/kg did not affect body weight (b.w.) nor accumulated weight change during the 28 day treatment period.

Group	Day 0	Day 7	Day 14	Day 21	Day 28
**Control**	**(*n* = 6)**	**(*n* = 6)**	**(*n* = 6)**	**(*n* = 6)**	**(*n* = 6)**
Body weight (g)	21.6 ± 0.8	22.3 ± 0.8	24.4 ± 1.0	24.9 ± 1.2	24.5 ± 1.4
Cumulative b.w. (g)	0.0	0.7 ± 0.5	2.8 ± 0.6	3.3 ± 0.4	2.9 ± 1.4
**20 µg/kg TTX**	**(*n* = 8)**	**(*n* = 8)**	**(*n* = 8)**	**(*n* = 8)**	(*n* = 7) *
Body weight (g)	23.4 ± 0.3	23.7 ± 0.7	24.7 ± 0.5	24.7 ± 0.9	26.1 ± 0.6
Cumulative b.w. (g)	0.0	0.3 ± 0.6	1.3 ± 0.5	2.1 ± 0.5	3.2 ± 0.6
**44 µg/kg TTX**	**(*n* = 10)**	**(*n* = 10)**	**(*n* = 10)**	**(*n* = 10)**	**(*n* = 10)**
Body weight (g)	24.8 ± 0.2	25 ± 1.0	26.0 ± 0.9	27.6 ± 1.0	29.0 ± 1.0
Cumulative b.w. (g)	0.0	0.2 ± 0.6	1.2 ± 0.5	2.8 ± 0.7	4.2 ± 1.0

Values are expressed as mean ± SEM. Data were registered weekly over the 28 days treatment period. The number of animals for each condition is indicated in parentheses. * One mouse died suddenly by accidental administration on the last day of treatment.

**Table 3:** Effect of daily oral administration of TTX on urine parameters of mice after the 28 day treatment period.

Group/Analyzed Parameters	(6) Control	(4) 20 µg/kg TTX	(10) 44 µg/kg TTX
**Color**	(6) Pale Yellow	(4) Dark Yellow	(7) Amber (3) Dark Yellow
**Turbidity**	(4) Clear (2) Cloudy	(4) Cloudy	(3) Slightly cloudy (2) Cloudy (5) Very cloudy
**Specific Gravity**	(1) 1020 (1) 1030 (4) >1050	(4) >1050	(1) 1016 (1) 1026 (1) 1030 (7) >1050
**Urine protein (g/L)**	Mean ± SEM: 1.26 ± 0.95 (1) Trace (2) 0 (1) 0.3 (1) 1 (1) 5	Mean ± SEM: 0.3 ± 0.2 (2) 0 (1) 0.3 (1) 1	Mean ± SEM: 0.3 ± 0.0 (1) Trace (9) 0
**Glucose (mmol/L)**	Mean ± SEM: 0.5 ± 0.5 (5) 0 (1) 3	Mean ± SEM: 3 ± 0.0 (4) 3	Mean ± SEM: 3.3 ± 3.0 (9) 3 (1) 6
**Ketones (mmol/L)**	Mean ± SEM: 0 (6) 0	Mean ± SEM: 0.8 ± 0.4 (2) 0 (2) 1.5	Mean ± SEM: 0 (10) 0
**Blood/Hemoglobin (Ery/µL)**	Mean ± SEM: 0 (6) 0	Mean ± SEM: 2.5 ± 2.5 (3) 0 (1) 10	Mean ± SEM: 4 ± 1.6 (6) 0 (4) 10
**Bilirubin (µmol/L)**	Mean ± SEM: 11.16 ± 8.25 (4) 0 (1) 17 (1) 50	Mean ± SEM: 8.5 ± 4.9 (2) 0 (2) 17	Mean ± SEM: 35.2 ± 8.7 (6) 17 (3) 50 (1) 100
**Urobilinogen (µmol/L)**	Mean ± SEM: 2.83 ± 2.83 (5) 0 (1) 17	Mean ± SEM: 8.5 ± 4.9 (2) 0 (2) 17	Mean ± SEM: 3.4 ± 2.3 (8) 0 (2) 17

On the day before sacrifice mice, with free access to food and water, were fed with TTX at doses of either 20 µg/kg or 44 µg/kg and urine was collected over the following 24 h. Control mice were fed with toxin solvent. Analytic parameters are expressed as mean ± SEM. The number of animals in each condition is shown in parenthesis.

**Table 4:** TTX determination in stomach, duodenum, and rectum.

*Samples*	TTX in Stomach (ng/g)		TTX in Duodenum (ng/g)		TTX in Rectum (ng/g)	
	Replica 1	Replica 2	Replica 1	Replica 2	Replica 1	Replica 2
Sample 1	12.4	14.2	13.1	14.9	n.d.	n.d.
Sample 2	n.d.	n.d.	n.d	n.d.	28.5	33.7
Sample 3	n.d.	n.d.	2.7	2.3	n.d.	n.d.

The term “n.d.” means not detected. LOD (organs) < 2.3 ng/g, LOD (blood) < 5.7 ng/g, LOD (feces and urine) < 3.3 ng/g.

**Table 5:** Neither control with solvent nor the combination of 5.3 µg/kg, 17 µg/kg and 54 µg/kg STX with 44 µg/kg TTX altered b.w. or accumulated weight change of mice during the 28 day treatment period.

Group	Day 0	Day 7	Day 14	Day 21	Day 28
**Control**	(*n* = 4)	(*n* = 4)	(*n* = 4)	(*n* = 4)	(*n* = 4)
Body weight (g)	21.4 ± 1.2	23.1 ± 1.4	24.6 ± 1.2	25.9 ± 1.5	25.1 ± 1.3
Cumulative b.w. (g)	0.0	1.7 ± 1.0	3.2 ± 0.8	2.9 ± 0.5	0.5 ± 0.5
**5.3 µg/kg STX + 44 µg/kg TTX**	(*n* = 4)	(*n* = 4)	(*n* = 4)	(*n* = 4)	(*n* = 3)
Body weight (g)	21.3 ± 0.6	24.0 ± 1.2	24.5 ± 1.2	25.6 ± 1.4	24.6 ± 2.4
Cumulative b.w. (g)	0.0	2.7 ± 1.0	3.2 ± 0.9	1.8 ± 0.7	−0.6 ± 1.2
**17 µg/kg STX + 44 µg/kg TTX**	(*n* = 5)	(*n* = 5)	(*n* = 5)	(*n* = 5)	(*n* = 5)
Body weight (g)	21.0 ± 1.5	22.5 ± 0.9	24.1 ± 0.8	24.9 ± 0.9	22.7 ± 1.0
Cumulative b.w. (g)	0.0	1.5 ± 0.8	3.1 ± 1.0	2.5 ± 0.3	−1.4 ± 0.4
**54 µg/kg STX + 44 µg/kg TTX**	(*n* = 5)	(*n* = 4)	(*n* = 3)	(*n* = 3)	(*n* = 3)
Body weight (g)	22.5 ± 0.3	23.4 ± 0.9	23.6 ± 0.7	24.0 ± 0.7	24.4 ± 0.9
Cumulative b.w. (g)	0.0	0.8 ± 0.7	1.2 ± 0.2	1.5 ± 0.2	0.7 ± 0.3

The number of animals for each condition is indicated in parenthesis. Values are expressed as mean ± SEM.

**Table 6:** Results obtained after chronic oral treatment for 28 days of mice with toxin solvent (control) and mice fed with 5.3 µg/kg, 17 µg/kg or 54 µg/kg STX combined with 44 µg/kg TTX regarding feed consumption, feces and urine production during 24 h prior sacrifice. Values are expressed as mean ± SEM.

Group/Analyzed Parameters (24 h in Metabolic Cages)	Title
**Control (*n* = 4)**	
Feed consumption (g)	4.4 ± 0.8
Feces (g)	1.2 ± 0.3
Urine (ml)	0.8 ± 0.2
**5.3 µg/kg STX + 44 µg/kg TTX (*n* = 3)**	
Feed consumption (g)	3.8 ± 1.1
Feces (g)	1.0 ± 0.4
Urine (ml)	0.4 ± 0.3
**17 µg/kg STX + 44 µg/kg TTX (*n* = 5)**	
Feed consumption (g)	2.5 ± 0.5
Feces (g)	1.0 ± 0.1
Urine (ml)	0.4 ± 0.1
**54 µg/kg STX + 44 µg/kg TTX (*n* = 3)**	
Feed consumption (g)	5.5 ± 0.6
Feces (g)	1.8 ± 0.1
Urine (ml)	1.2 ± 0.3

**Table 7:** Precursor and product ions (*m*/*z)* TTX analogues and MRM conditions.

Toxins	Precursor Ion	Product Ion	CE	Frag	CAV	Polarity
11-norTTX-6(R/S)-ol	290	272	24	152	2	Positive
		162	40			
11-oxo-TTX	336	162	40	152	2	Positive
		318	24			
1-hydroxy-8-epi-5,6,11-trideoxyTTX	288	162	40	152	2	Positive
		270	24			
4,9-anhydroTTX 6-epi-4,9-anhydroTTX	302	162	40	152	4	Positive
		256	28			
4-anhydro-8-epi-5,6,11-trideoxyTTX	254	162	40	152	2	Positive
		236	24			
5,6,11-trideoxyTTX 8-epi-5,6,11-trideoxyTTX	272	162	40	152	2	Positive
		254	24			
5-deoxyTTX 11-deoxyTTX	304	176	40	152	2	Positive
		286	24			
6,11-dideoxyTTX	288	224	40	152	2	Positive
		270	24			
TTX; TDA 4-epiTTX; 6-epiTTX	320	162	36	160	2	Positive
		302	24			

## References

[1] Bane V., Lehane M., Dikshit M., O’Riordan A., Furey A. (2014). Tetrodotoxin: Chemistry, toxicity, source, distribution and detection. Toxins.

[2] Katikou P., Georgantelis D., Sinouris N., Petsi A., Fotaras T. (2009). First report on toxicity assessment of the Lessepsian migrant pufferfish *Lagocephalus sceleratus* (Gmelin, 1789) from European waters (Aegean Sea, Greece). Toxicon.

[3] Vlamis A., Katikou P., Rodriguez I., Rey V., Alfonso A., Papazachariou A., Zacharaki T., Botana A.M., Botana L.M. (2015). First detection of tetrodotoxin in greek shellfish by UPLC-MS/MS potentially linked to the presence of the dinoflagellate *Prorocentrum minimum*. Toxins.

[4] Almeida P., Diaz R., Hernandez F., Ferrer G. (2019). Blow: A case of pufferfish intoxication in South Florida. BMJ Case Rep..

[5] Rodriguez P., Alfonso A., Vale C., Alfonso C., Vale P., Tellez A., Botana L.M. (2008). First toxicity report of tetrodotoxin and 5,6,11-TrideoxyTTX in the trumpet shell *Charonia lampas lampas* in Europe. Anal. Chem..

[6] Fernandez-Figares M., Fernandez V., Postigo M.J., Feron P. (2013). Acute paralysis after seafood ingestion. Neurophysiol. Clin..

[7] Fernandez-Ortega J.F., Morales-de los Santos J.M., Herrera-Gutierrez M.E., Fernandez-Sanchez V., Rodriguez Loureo P., Rancano A.A., Tellez-Andrade A. (2010). Seafood intoxication by tetrodotoxin: First case in Europe. J. Emerg. Med..

[8] Rambla-Alegre M., Reverte L., Del Rio V., de la Iglesia P., Palacios O., Flores C., Caixach J., Campbell K., Elliott C.T., Izquierdo-Munoz A. (2017). Evaluation of tetrodotoxins in puffer fish caught along the Mediterranean coast of Spain. Toxin profile of *Lagocephalus sceleratus*. Environ. Res..

[9] Leonardo S., Kiparissis S., Rambla-Alegre M., Almarza S., Roque A., Andree K.B., Christidis A., Flores C., Caixach J., Campbell K. (2019). Detection of tetrodotoxins in juvenile pufferfish Lagocephalus sceleratus (Gmelin, 1789) from the North Aegean Sea (Greece) by an electrochemical magnetic bead-based immunosensing tool. Food Chem..

[10] Katikou P. (2019). Public health risks associated with tetrodotoxin and its analogues in european waters: Recent advances after the EFSA scientific opinion. Toxins.

[11] Turner A.D., Powell A., Schofield A., Lees D.N., Baker-Austin C. (2015). Detection of the pufferfish toxin tetrodotoxin in European bivalves, England, 2013 to 2014. Eurosurveillance.

[12] Leao J.M., Lozano-Leon A., Giraldez J., Vilarino O., Gago-Martinez A. (2018). Preliminary results on the evaluation of the occurrence of tetrodotoxin associated to marine vibrio spp. in bivalves from the galician rias (Northwest of Spain). Mar. Drugs.

[13] Rodrigues S., Pinto E., Oliveira P., Pedro S., Reis Costa P. (2019). Evaluation of the occurrence of tetrodotoxin in bivalve mollusks from the portuguese coast. J. Mar. Sci. Eng..

[14] Pinto E.P., Rodrigues S.M., Gouveia N., Timoteo V., Costa P.R. (2019). Tetrodotoxin and saxitoxin in two native species of puffer fish, Sphoeroides marmoratus and Lagocephalus lagocephalus, from NE Atlantic Ocean (Madeira Island, Portugal). Mar. Environ. Res..

[15] Dell’Aversano C., Tartaglione L., Polito G., Dean K., Giacobbe M., Casabianca S., Capellacci S., Penna A., Turner A.D. (2019). First detection of tetrodotoxin and high levels of paralytic shellfish poisoning toxins in shellfish from Sicily (Italy) by three different analytical methods. Chemosphere.

[16] Vilarino N., Louzao M.C., Abal P., Cagide E., Carrera C., Vieytes M.R., Botana L.M. (2018). Human Poisoning from Marine Toxins: Unknowns for Optimal Consumer Protection. Toxins.

[17] Finch S.C., Boundy M.J., Harwood D.T. (2018). The Acute Toxicity of Tetrodotoxin and Tetrodotoxin(-)Saxitoxin Mixtures to Mice by Various Routes of Administration. Toxins.

[18] European Union E. (2004). Corrigendum to Regulation (EC) No 853/2004 of the European Parliament and of the Council of 29 April 2004 laying down specific hygiene rules for food of animal origin. Off. J. Eur. Union.

[19] Gerssen A., Bovee T., Klijnstra M., Poelman M., Portier L., Hoogenboom R. (2018). First report on the occurrence of tetrodotoxins in bivalve mollusks in The Netherlands. Toxins.

[20] Knutsen H., Alexander J., Barreg ard L., Bignami M., Brüschweiler B., Ceccatelli S., Cottrill B., Dinovi M., Edler L., Grasl-Kraupp B. (2017). Risks for public health related to the presence of tetrodotoxin (TTX) and TTX analogues in marine bivalves and gastropods. EFSA J..

[21] Abal P., Louzao M.C., Antelo A., Alvarez M., Cagide E., Vilarino N., Vieytes M.R., Botana L.M. (2017). Acute oral toxicity of tetrodotoxin in mice: Determination of Lethal Dose 50 (LD50) and No Observed Adverse Effect Level (NOAEL). Toxins.

[22] Abal P., Louzao M.C., Vilarino N., Vieytes M.R., Botana L.M. (2019). Acute toxicity assessment: Macroscopic and ultrastructural effects in mice treated with oral tetrodotoxin. Toxins.

[23] Boente-Juncal A., Vale C., Cifuentes M., Otero P., Camina M., Rodriguez-Vieytes M., Botana L.M. (2019). Chronic in vivo effects of repeated exposure to low oral doses of tetrodotoxin: Preliminary evidence of nephrotoxicity and cardiotoxicity. Toxins.

[24] EFSA (2009). Panel on contaminants in the food chain (CONTAM). Scientific opinion on marine biotoxins in shellfish—Saxitoxin group. EFSA J..

[25] OECD (2008). Test No. 407: Repeated Dose 28-day Oral Toxicity Study in Rodents.

[26] Catterall W.A. (2015). Finding channels. J. Biol. Chem..

[27] Rodriguez I., Alfonso A., Gonzalez-Jartin J.M., Vieytes M.R., Botana L.M. (2018). A single run UPLC-MS/MS method for detection of all EU-regulated marine toxins. Talanta.

[28] Tamele I.J., Silva M., Vasconcelos V. (2019). The incidence of tetrodotoxin and its analogs in the Indian Ocean and the red sea. Mar. Drugs.

[29] Botana L.M. (2016). Toxicological perspective on climate change: Aquatic toxins. Chem. Res. Toxicol..

[30] Silva M., Pratheepa V.K., Botana L.M., Vasconcelos V. (2015). Emergent toxins in North Atlantic temperate waters: A challenge for monitoring programs and legislation. Toxins.

[31] Silva M., Rodriguez I., Barreiro Felpeto A., Kaufmann M., Neto A.I., Hassouani M., Sabour B., Alfonso A., Botana L., Vasconcelos V. (2019). Tetrodotoxins occurrence in non-traditional vectors of the north atlantic waters (portuguese maritime territory, and morocco coast). Toxins.

[32] Lago J., Rodríguez L.P., Blanco L., Vieites J.M., Cabado A.G. (2015). Tetrodotoxin, an extremely potent marine neurotoxin: Distribution, toxicity, origin and therapeutical uses. Mar. Drugs.

[33] Zepeda R.J., Candiracci M., Lobos N., Lux S., Miranda H.F. (2014). Chronic toxicity study of neosaxitoxin in rats. Mar. Drugs.

[34] Zimmer T. (2010). Effects of tetrodotoxin on the mammalian cardiovascular system. Mar. Drugs.

[35] Chowdhury F.R., Ahasan H.A., Al Mamun A., Rashid A.K., Al Mahboob A. (2007). Puffer fish (Tetrodotoxin) poisoning: An analysis and outcome of six cases. Trop. Dr..

[36] How C.K., Chern C.H., Huang Y.C., Wang L.M., Lee C.H. (2003). Tetrodotoxin poisoning. Am. J. Emerg. Med..

[37] Hwang D.F., Noguchi T. (2007). Tetrodotoxin Poisoning. Advances in Food and Nutrition Research.

[38] Noguchi T., Onuki K., Arakawa O. (2011). Tetrodotoxin poisoning due to pufferfish and gastropods, and their intoxication mechanism. ISRN Toxicol..

[39] Cheng H.S., Chua S.O., Hung J.S., Yip K.K. (1991). Creatine kinase MB elevation in paralytic shellfish poisoning. Chest.

[40] Brewster L.M., Karamat F.A., van Montfrans G.A. (2019). Creatine kinase and blood pressure: A systematic review. Med. Sci..

